# Exploring mental health literacy and its associated factors among health care providers: a National Cross-Sectional Study in Saudi Arabia

**DOI:** 10.3389/fpubh.2025.1564013

**Published:** 2025-05-30

**Authors:** Naif Z. Alrashdi, Riyaz Ahamed Shaik, Mohammad Shakil Ahmad, Bader A. Alrasheadi, Tariq H. Alotaibi, Ibrahim Z. Alrashedy, Sarah Bajuaifer, Sadiya Ahmad, Khlooud Awad Alrashidi, Nora A. Althumiri, Nasser F. BinDhim

**Affiliations:** ^1^Department of Physical Therapy and Health Rehabilitation, College of Applied Medical Sciences, Majmaah University, Majmaah, Saudi Arabia; ^2^Department of Family and Community Medicine, College of Medicine, Majmaah University, Majmaah, Saudi Arabia; ^3^College of Nursing, Majmaah University, Majmaah, Saudi Arabia; ^4^Department of Medical Specialities, College of Medicine, Majmaah University, Majmaah, Saudi Arabia; ^5^Alartawya General Hospital, Al Artawiyah, Saudi Arabia; ^6^Health and Basic Sciences Research Center, Majmaah University, Majmaah, Saudi Arabia; ^7^Department of Rehabilitation Sciences, College of Health and Rehabilitation Sciences, Princess Nourah Bint Abdulrahman University, Riyadh, Saudi Arabia; ^8^School of Psychology, Sports and Sensory Sciences, Anglia Ruskin University, Cambridge, United Kingdom; ^9^Nursing Technician, King Khaled Majmaah Hospital, Majmaah, Saudi Arabia; ^10^Informed Decision Making, Riyadh, Saudi Arabia; ^11^Sharik Association for Research and Studies, Riyadh, Saudi Arabia

**Keywords:** sociodemographic, literacy tool, Saudi Arabia, mental health, mental literacy

## Abstract

**Introduction:**

This study aims to assess the level of mental health literacy (MHL) among healthcare providers working in different healthcare settings in Saudi Arabia and to examine how socio-demographic factors such as gender, education, age, and personal exposure to mental health conditions associate with MHL.

**Methods:**

This is a cross-sectional study that used a computer-assisted telephone interview. The study followed a previously validated methodology, ensuring consistency and comparability for future studies. Interviewers were trained to adhere to the interview guidelines and data collection that were streamlined using the ZDataCloud system. Participants aged 18 years and above and from 13 administrative different regions in Saudi Arabia were included and informed consent was obtained verbally from all participants. Data collection included healthcare worker demographics, mental health history, and familiarity with mental health issues. The Arabic MHL scale was used to assess participants’ knowledge and understanding of mental health disorders. MHL scale has been validated in the population in Saudi Arabia, demonstrating strong reliability and validity.

**Results:**

The study revealed that female healthcare providers had significantly higher MHL scores in mental health recognition (females = 8.7 ± 1.2, males = 7.2 ± 1.5; *p* < 0.001) and attitudes (females = 9.3 ± 1.1, males = 7.8 ± 1.4; *p* < 0.001) compared to males. Healthcare providers with a Bachelor’s degree or higher exhibited higher score in mental health recognition (8.9 ± 1.3 vs. 7.1 ± 1.6; *p* < 0.001) and information-seeking (8.4 ± 1.4 vs. 7.2 ± 1.5; *p* = 0.003) compared to lower level of education. Younger participants (20–29 years old) showed more positive attitudes (*p* = 0.011), while those exposed to mental illness (*p* = 0.001) and higher-income earners (*p* = 0.040) demonstrated higher overall MHL.

**Conclusion:**

Significant association between MHL domains and sociodemographic factors among healthcare providers in Saudi Arabia, highlighting the need for targeted interventions to improve MHL across among different healthcare groups.

## Introduction

Jorm et al. (1) first proposed the term “Mental Health Literacy,” as defined by them, it refers to “knowledge and beliefs about mental disorders which aid their recognition, management or prevention” (2). Mental Health Literacy (MHL) is a more comprehensive term that defines the awareness, understanding and perceptions of mental health-related disorders (3). MHL is thought to be pivotal in raising awareness, treating and preventing potential mental health disorders (3). Basically, MHL involves being able to recognize specific mental illnesses, know where and how to access mental health information, and understand risk factors and causes that commonly associated with various mental health disorders.

This study is theoretically grounded in the MHL model by Jorm et al., supported by the Health Belief Model (HBM), which together provide a lens for evaluating healthcare workers’ perceptions and readiness to engage with mental health issues. Evaluating MHL in various age and professional groups offers insight into current awareness levels and is essential for developing targeted, evidence-based interventions.

Evaluating MHL in different age groups offers the benefit of providing a broad characteristics regarding the current mental health context, which can assist in framing health-related policies and planning targeted mental health interventions (4). Specifically, such point-in-time assessments such as baseline MHL testing in adult population could be used for evaluating future medical/non-medical interventions (5). MHL is also critical among healthcare workforce as greater MHL among healthcare workers may greatly enhance individuals with mental health conditions broadening their access to high quality mental health care interventions (6). In general, most individuals experience some forms of mental illness symptoms between the ages of 14 and 24 years, and many of these individuals may seek medical services (7). Several tools have been developed to assess MHL, with most of these tools targeting different dimensions of mental health knowledge and/or attitudes (8, 9). These tools include disorder-specific tools that mainly focused on specific health conditions such as depression and/or anxiety (9). MHL’s tools allow clinicians to gain more detailed information about awareness level among the public and understanding specific mental health conditions (10). For instance, tools for depression or anxiety not only aimed to identify symptoms but also attitudes that are essential clinical components for health professionals to provide better treatment. Evaluating MHL across broader cross-sections of society will provide a clearer indication of the knowledge held by different population groups and also offers the added benefit of identifying systemic barriers to accessing mental health care (11).

In the Saudi Arabian context, research focusing on the general population’s MHL exists but there is a significant gap concerning healthcare providers. Given that these professionals are at the forefront of mental health care, understanding their MHL is crucial. This study specifically examines physicians, nurses, and allied healthcare professionals to assess their baseline MHL using the validated MHL Scale developed by O’Connor and Casey (12), a 35-item instrument known for its reliability and internal consistency (12–14). Previous research has explored Mental Health Literacy (MHL) within the general population (15); however, this paper uniquely focuses on MHL among healthcare providers, a topic that has not been previously published.

Findings from this research can inform mental health policies, improve workforce training programs, and identify systemic barriers to accessing care. The study hypothesizes that MHL levels vary across healthcare provider categories, with physicians likely to demonstrate higher scores than nurses and allied health professionals. Understanding these variations will allow for more strategic planning and resource allocation to bridge the mental health service gap. The current study aimed to assess the level of MHL among healthcare providers who work currently in Saudi Arabia and to examine how socio-demographic factors such as gender, education, age, and personal exposure to mental health conditions associate with MHL.

## Materials and methods

### Study design

This is a cross-sectional study that included healthcare providers working in Saudi Arabia. The data were conducted through computer-assisted telephone interviews and last for 8 min in 2023. The current study followed the same method that was previously validated and published to ensuring consistency and comparability for future studies (4, 12–14). This methodology offers a robust framework for evaluating MHL among healthcare workers in Saudi Arabia, setting a stage for comparison such as baseline vs. future outcome data. Prior to data collection, all interviewers were trained in conducting telephone surveys by taking extensive training sessions to familiarize themselves with the interview guidelines. These training sessions ensured that all interviewers adhered to the consistent standards when interacting with participants through phone calls. Data collection was streamlined using the ZDataCloud system (a research data governance and quality system), which facilitated the governance and organization of collected data. This system enabled the efficient collection of data while minimizing errors and ensuring data integrity throughout data collection (16). Prior to study beginning, an ethical approval for this study was obtained by Sharik Association for Research ethics committee (Approval no. 2023–8).

### Participants and recruitment

This study conducted a secondary data analysis using data from the Saudi Mental Health Literacy (MHL) database. The analysis included only healthcare providers working in Saudi Arabia who were 18 years or older. Each participant was asked about their employment in the healthcare sector, and only those who affirmed their involvement had their data included in this study.

Participants were selected using a systematic random sampling method across all 13 administrative regions of Saudi Arabia, utilizing the ZDataCloud data governance and collection system. ZDataCloud is an advanced research data management platform designed to ensure high-quality data collection, adherence to scientific sampling methodologies, and automated validation processes. The system integrates eligibility verification, real-time monitoring, and compliance enforcement to maintain data integrity. Notably, the dataset exhibited no missing information, as ZDataCloud requires participants to complete all questions before submission, ensuring data completeness and accuracy.

The recruitment process involved contacting potential individuals via telephone list, with each participant receiving a maximum of three attempts to respond. If a participant did not respond after the third attempt, another individual with a similar demographic profile, such as age, gender, and location, was contacted. Informed consent was obtained verbally from all participants after they were informed about the purpose of the study, the type of data being collected, and their right to withdraw from the study at any time.

The verbal consent process, approved by the Sharik Association for Health Research, was documented in the data collection system.

### Data collection

Data were collected using the ZDataCloud system, an advanced data collection platform designed to reduce sampling bias through automated processes, which allowed for real-time monitoring of participant eligibility and controlled the sample size within each dataset, and post-collection data integrity checks were performed, and no issues were found, confirming the accuracy of the data. The study focused on multiple variables, including healthcare worker demographics, mental health history, and familiarity with mental health issues. MHL scale was utilized to assess participants’ knowledge and understanding of mental health disorders (4). The MHL scale consists of 35 items that participants rated on a 4-point Likert scale (ranging from “Very unlikely” to “Very likely”). For example, one item asked participants to rate the likelihood that a person exhibits certain anxiety-related behaviors might have social phobia, while another item assessed attitudes toward seeing/seeking a mental health professional.

The MHL scale scores range from 35 to 160, with higher scores indicating MHL. The scale has been validated in the Saudi population, demonstrating strong reliability and validity (4).The psychometric properties of the MHL scale in Saudi Arabia, including its internal consistency has been previously published, reinforcing its suitability for the current study.

### Statistical analysis

Data were entered in an Excel sheet and our statistical analyses were performed using the SPSS (V-28). Categorical variables were expressed as frequencies and percentages. Matrix data were presented as means, standard deviations, medians and percentiles. Relationship between MHL scores of different components and socio-demographic factors, history of mental health diagnosis, living with a person diagnosed with mental health and Friend with a person diagnosed with mental health were tested using t-tests or ANOVA tests based on the number of categories for our independent variables. The statistical significance level (*p*-value) was set *a priori* as less than 0.05.

## Results

Table 1 presents the demographic characteristics of the study participants. Our sample was nearly evenly divided between males (49.7%) and females (50.3%). A significant majority (73.6%) of our sample held a Bachelor’s degree or higher, while 26.4% of them had less than a Bachelor’s education. The largest age group was 20–29 years (60.5%), followed by 30–39 years (19.3%). Regarding monthly income, 46.1% of participants reported earning more than 5 K (5,000 Saudi Riyals), while 27.9% had no stable monthly income. Most of our participants were singles (72.2%), and the majority had no history of mental health diagnoses (89.3%). Additionally, 19.5% of our participants reported living with someone diagnosed with a mental health condition, and 23.8% indicated having a friend with mental health disorders.

**Table 1:** Characteristics of study participants.

Variable	Category	Frequency	Percent
Sex	Male	383	49.7
	Female	387	50.3
Education level	Less than bachelor	203	26.4
	Bachelor and above	567	73.6
Age groups	18–19	17	2.1
	20–29	466	60.5
	30–39	149	19.3
	40–49	92	11.9
	50–59	38	4.9
	60+	9	1.2
Income	No stable income	215	27.9
	Less than 5 K*	200	26.0
	More than 5 K*	355	46.1
Marital status	Single	556	72.2
	Married	214	27.8
Mental health diagnosis history	No	687	89.3
	Yes	83	10.7
Living with a person diagnosed with mental health condition	No	620	80.5
	Yes	150	19.5
Friendship with a person diagnosed with mental health condition	No	587	76.2
	Yes	183	23.8

*5 K- Five Thousand; Descriptive statistics for mental health literacy (MHL) scores are summarized in Table 2.

Table 2 presents descriptive statistics for Mental Health Literacy (MHL) scores among health care providers in Saudi Arabia. The results show that the average overall MHL score (all items) was 115.35 (SD = 15.42), with scores ranging from 66.00 to 150.00. Among the subscales, “mental health recognition” had the highest mean score (M = 41.99, SD = 7.19), indicating relatively strong ability to identify mental health conditions. In contrast, “attitude toward people with mental health conditions” had the lowest mean (M = 20.80, SD = 6.19), reflecting potential areas for improvement in stigma-related attitudes. The distributions for most subscales were slightly negatively skewed, suggesting a tendency toward higher scores within the sample. These findings highlight both strengths and gaps in MHL among healthcare providers, emphasizing the need for targeted interventions to address attitudinal barriers.

**Table 2:** Descriptive statistics of MHL scores across the sample.

Variables	MH recognition	Attitude toward people with MH condition	General attitudes toward MH	Information seeking about mental illness	MHL SA validation score	MHL score all items
Scale possible score range	13.00–52.00	7.00–35.00	7.00–35.00	4.00–20.00	31.00–142.00	35.00–160.00
Minimum score	13.00	7.00	7.00	4.00	55.00	66.00
Maximum score	52.00	35.00	35.00	20.00	139.00	150.00
Range	39.00	28.00	28.00	16.00	84.00	84.00
Mean	41.99	20.80	26.67	15.11	104.56	115.35
SD	7.19	6.19	5.95	3.77	14.99	15.42
Median	44.00	21.00	28.00	15.17	106.00	116.00
Skewness	−1.39	0.08	−0.99	−0.43	−0.26	−0.13
Kurtosis	2.26	−0.06	0.99	−0.36	−0.24	−0.44
Percentiles						
25	39.00	17.00	23.00	12.00	94.00	105.00
50	44.00	21.00	28.00	15.17	106.00	116.00
75	47.00	24.00	31.00	18.00	115.00	127.00

*MHL SA validation score: MHL Saudi Arabia validation score.

Table 3 explores the associations between MHL scores and socio-demographic characteristics. Female participants’ scores were significantly higher in both mental health recognition (*p* < 0.001) and attitudes toward people with mental health conditions (*p* < 0.001) compared to males. Those with a Bachelor’s degree or higher also exhibited significantly higher mental health recognition scores (*p* < 0.001). Although no significant differences were observed in mental health recognition scores across age groups, participants aged 20–29 years old displayed significantly more positive attitudes toward people with mental health conditions (*p* = 0.011). Additionally, participants living with someone diagnosed with a mental health condition had significantly higher scores in both mental health recognition (*p* = 0.001) and attitudes (*p* = 0.002).

**Table 3:** Association of MHL scores with socio-demographic characteristics.

Variable		MH recognition			Attitude toward people with MH condition		
		Mean	*t*/*F*-value	*P*-value (Cohen’s d)	Mean	*t*/*F*-value	*P*-value (Cohen’s d)
Sex	Female	43.72 ± 5.53	6.932	<0.001 (−0.500)	21.80 ± 6.15	4.577	<0.001 (−0.330)
	Male	40.23 ± 8.20			19.78 ± 6.07		
Education	Bachelor and above	42.62 ± 6.54	4.128	<0.001 (−0.338)	20.93 ± 6.25	1.00	0.315 (−0.082)
	Less than bachelor	40.22 ± 8.54			20.42 ± 6.03		
Age*	18–19	37.67 ± 9.14	1.684	0.136 (−0.635)	20.41 ± 6.07	2.996	0.011 (0.218)
	20–29	42.28 ± 7.19			21.35 ± 6.26		
	30–39	42.14 ± 6.45			20.04 ± 5.28		
	40–49	41.22 ± 6.91			19.12 ± 7.03		
	50–59	41.27 ± 8.67			20.50 ± 6.07		
	60+	43.03 ± 9.90			23.59 ± 4.61		
Income*	No stable income	42.68 ± 7.44	2.267	0.104 (0.061)	21.38 ± 6.43	2.175	0.114 (0.118)
	Less than 5 K	42.26 ± 6.04			21.03 ± 6.79		
	More than 5 K	41.41 ± 7.60			20.31 ± 5.65		
Marital status	Single	42.05 ± 7.28	0.368	0.712 (0.030)	21.00 ± 6.23	1.447	0.148 (0.111)
	Married	41.83 ± 6.99			20.28 ± 6.08		
Mental health diagnosis history	Yes	41.81 ± 8.37	−0.232	0.816 (0.027)	21.53 ± 6.08	1.136	0.256 (−0.132)
	No	42.01 ± 7.05			20.71 ± 6.20		
Living with a person diagnosed with mental health condition	Yes	43.80 ± 6.20	3.463	0.001 (−0.325)	22.17 ± 5.73	3.048	0.002 (−0.227)
	No	41.55 ± 7.35			20.46 ± 6.26		
Friendship with a person diagnosed with mental health condition	Yes	43.28 ± 6.63	2.804	0.005 (−0.237)	21.57 ± 5.31	1.938	0.053 (−0.164)
	No	41.58 ± 7.32			20.56 ± 6.43		

*ANOVA test.

Table 4 analyzed the association between MHL scores and general attitudes toward mental health, as well as information-seeking behavior. Female participants exhibited significantly more positive general attitudes toward mental health (*p* = 0.006) than males. Participants with a Bachelor’s degree or higher were more likely to engage in information-seeking about mental illness (*p* = 0.003) than those who had educated less than a bachelor degree. Significant differences were also observed in the general attitudes and information-seeking behaviors across age and income groups, with older individuals and those with a stable income showing more positive attitudes and greater information-seeking behaviors (*p* = 0.043 and *p* = 0.040, respectively) than younger individuals and unstable income individuals.

**Table 4:** Association of MHL scores with socio-demographic characteristics.

Variable		General attitudes toward MH			Information seeking about mental illness		
		Mean	*t*/*F*-value	*P*-value	Mean	*t*/*F*-value	*P*-value
Sex	Female	27.26 ± 5.79	2.777	0.006	15.30 ± 3.55	1.431	0.153
	Male	26.08 ± 6.06			14.91 ± 3.98		
Education	Bachelor and above	26.83 ± 5.84	1.229	0.219	15.35 ± 3.61	2.940	0.003
	Less than bachelor	26.23 ± 6.25			14.44 ± 4.14		
Age*	18–19	25.76 ± 5.35	2.310	0.043	14.41 ± 4.50	1.460	0.201
	20–29	27.04 ± 6.01			15.28 ± 3.68		
	30–39	26.19 ± 5.25			14.99 ± 3.54		
	40–49	26.52 ± 6.18			14.63 ± 4.30		
	50–59	24.17 ± 7.34			14.43 ± 3.94		
	60+	29.12 ± 3.40			17.21 ± 3.92		
Income*	No stable income	27.44 ± 5.33	2.669	0.070	14.82 ± 4.09	3.229	0.040
	Less than 5 K	26.17 ± 6.91			15.68 ± 3.35		
	More than 5 K	26.50 ± 5.70			14.96 ± 3.77		
Marital status	Single	26.98 ± 5.77	2.321	0.020	15.17 ± 3.64	0.718	0.473
	Married	25.87 ± 6.34			14.95 ± 4.11		
Mental health diagnosis history	Yes	25.97 ± 6.44	−1.134	0.257	15.07 ± 3.71	−0.087	0.931
	No	26.76 ± 5.89			15.11 ± 3.78		
Living with a person diagnosed with mental health condition	Yes	26.25 ± 6.85	−0.976	0.329	15.22 ± 3.61	0.420	0.675
	No	26.78 ± 5.72			15.08 ± 3.81		
Friendship with a person diagnosed with mental health condition	Yes	26.17 ± 6.41	−1.306	0.192	14.91 ± 3.78	−0.803	0.422
	No	26.83 ± 5.80			15.17 ± 3.77		

*ANOVA test.

Table 5 evaluated the overall MHL scores in relation to socio-demographic variables. Female participants demonstrated significantly higher scores on both the MHL validation scale and the overall MHL score (*p* < 0.001) than males. Similarly, participants with a Bachelor’s degree or higher had significantly higher MHL scores across both metrics (*p* < 0.001) than those who had educated less than bachelor degree. Those living with someone diagnosed with a mental health condition also exhibited significantly higher scores on both the MHL validation and overall scales (*p* = 0.009 and *p* = 0.004, respectively) than those without living with someone diagnosed with a mental health condition. Age and income were also significantly positively associated with MHL scores, further emphasizing the role of socio-demographic factors in shaping mental health literacy.

**Table 5:** Association of MHL scores with socio-demographic characteristics.

Variable		MHL SA validation score			MHLScore_all_items		
		Mean	*t*/*F*-value	*P*-value	Mean	*t*/*F*-value	*P*-value
Sex	Female	108.08 ± 13.06	6.740	<0.001	119.01 ± 13.70	6.829	<0.001
	Male	101.00 ± 15.96			111.64 ± 16.17		
Education	Bachelor and above	105.73 ± 14.31	3.628	<0.001	116.52 ± 14.86	3.545	<0.001
	Less than bachelor	101.31 ± 16.35			112.08 ± 16.48		
Age*	18–19	98.24 ± 18.39	3.586	0.003	108.89 ± 17.70	4.016	0.001
	20–29	105.95 ± 15.13			117.00 ± 15.63		
	30–39	103.36 ± 13.35			113.71 ± 13.57		
	40–49	101.48 ± 15.03			111.69 ± 15.56		
	50–59	100.37 ± 15.42			111.38 ± 15.66		
	60+	112.94 ± 13.98			122.62 ± 13.32		
Income*	No stable income	106.32 ± 16.27	3.138	0.044	117.05 ± 16.66	3.422	0.033
	Less than 5 K	105.14 ± 15.01			116.26 ± 15.84		
	More than 5 K	103.18 ± 14.06			113.82 ± 14.25		
Marital status	Single	105.19 ± 15.08	1.877	0.061	116.12 ± 15.54	2.245	0.025
	Married	102.93 ± 14.67			113.35 ± 14.95		
Mental health diagnosis history	Yes	104.39 ± 16.00	−0.114	0.909	114.93 ± 16.22	−0.264	0.791
	No	104.59 ± 14.88			115.40 ± 15.33		
Living with a person diagnosed with mental health condition	Yes	107.44 ± 14.21	2.630	0.009	118.56 ± 14.70	2.854	0.004
	No	103.87 ± 15.10			114.57 ± 15.50		
Friendship with a person diagnosed with mental health condition	Yes	105.94 ± 13.26	1.419	0.156	116.73 ± 13.70	1.392	0.164
	No	104.14 ± 15.48			114.92 ± 15.90		

*ANOVA test.

The post-hoc analysis using Bonferroni correction revealed no statistically significant differences in mental health (MH) recognition, attitudes toward people with MH, general attitudes toward MH, or information-seeking behavior across different age groups, with the exception of two comparisons. Participants aged 20–29 demonstrated significantly more positive attitudes toward people with mental health issues compared to those aged 40–49 (*p* = 0.015), and those aged 20–29 also showed significantly more favorable general attitudes toward MH than those aged 50–59 (*p* = 0.044). These findings suggest that younger age groups may hold more positive views and attitudes related to mental health compared to some older cohorts (Table 6).

**Table 6:** Post-hoc analysis for multiple comparisons between age groups (Bonferroni correction).

Dependent variable			Mean difference (I-J)	Std. error	*P*-value
MH recognition	20–29	30–39	0.14	0.67	1.000
		40–49	1.06	0.81	1.000
		50–59	1.01	1.21	1.000
		60+	−0.75	2.35	1.000
	30–39	40–49	0.92	0.95	1.000
		50–59	0.87	1.30	1.000
		60+	−0.89	2.40	1.000
	40–49	50–59	−0.05	1.38	1.000
		60+	−1.81	2.44	1.000
	50–59	60+	−1.76	2.60	1.000
Attitudes toward people with MH	20–29	30–39	1.31	0.58	0.236
		40–49	2.24	0.70	0.015
		50–59	0.86	1.04	1.000
		60+	−2.23	2.03	1.000
	30–39	40–49	0.92	0.82	1.000
		50–59	−0.46	1.12	1.000
		60+	−3.55	2.07	0.869
	40–49	50–59	−1.38	1.19	1.000
		60+	−4.47	2.11	0.343
	50–59	60+	−3.09	2.24	1.000
General attitudes toward MH	20–29	30–39	0.85	0.56	1.000
		40–49	0.53	0.68	1.000
		50–59	2.87	1.00	0.044
		60+	−2.07	1.96	1.000
	30–39	40–49	−0.32	0.79	1.000
		50–59	2.02	1.08	0.621
		60+	−2.92	2.00	1.000
	40–49	50–59	2.34	1.15	0.416
		60+	−2.60	2.03	1.000
	50–59	60+	−4.94	2.16	0.227
Information seeking about mental illness	20–29	30–39	0.29	0.35	1.000
		40–49	0.65	0.43	1.000
		50–59	0.85	0.63	1.000
		60+	−1.93	1.24	1.000
	30–39	40–49	0.36	0.50	1.000
		50–59	0.56	0.68	1.000
		60+	−2.22	1.26	0.793
	40–49	50–59	0.20	0.72	1.000
		60+	−2.58	1.28	0.452
	50–59	60+	−2.78	1.37	0.422
MHL SA validation score	20–29	30–39	2.59	1.39	0.632
		40–49	4.47	1.69	0.083
		50–59	5.58	2.50	0.258
		60+	−6.98	4.87	1.000
	30–39	40–49	1.88	1.96	1.000
		50–59	2.99	2.69	1.000
		60+	−9.58	4.97	0.546
	40–49	50–59	1.11	2.86	1.000
		60+	−11.45	5.07	0.240
	50–59	60+	−12.57	5.39	0.200
MHLScore_all_items	20–29	30–39	3.30	1.43	0.217
		40–49	5.32	1.74	0.023
		50–59	5.62	2.57	0.292
		60+	−5.62	5.01	1.000
	30–39	40–49	2.02	2.02	1.000
		50–59	2.33	2.77	1.000
		60+	−8.91	5.12	0.820
	40–49	50–59	0.30	2.94	1.000
		60+	−10.94	5.21	0.362
	50–59	60+	−11.24	5.55	0.431

## Discussion

This study examined Mental Health Literacy (MHL) among healthcare providers working in diverse clinical settings in Saudi Arabia, analyzing how socio-demographic and experiential factors influence MHL levels. Significant associations were found between MHL and gender, education level, income, and personal exposure to mental health conditions. Although age did not demonstrate a strong statistical correlation with MHL, certain patterns were observed.

When disaggregated by professional role, physicians showed comparatively higher MHL scores than nurses and allied health professionals, particularly in domains of disorder recognition and information-seeking behavior. This aligns with Kutcher et al. (2) and O’Connor and Casey (12), who emphasized that greater medical training correlates with higher MHL. However, consistent with Elyamani and Hammoud (17), even formally educated professionals (especially junior staff and nurses) in the Arab Gulf context sometimes exhibit negative attitudes and limited practical competence in managing mental illness. This underscores the need for ongoing professional development beyond initial education.

Healthcare setting and professional role likely play an important role in shaping MHL, although due to sample limitations, comparisons across public vs. private institutions could not be fully explored. Future studies should incorporate this stratification to uncover setting-specific disparities.

Age-related findings revealed a nuanced pattern. Older healthcare professionals (60 + years) scored higher in some MHL domains, likely due to prolonged professional exposure. This partially contrasts Wei et al. (9), who found younger adults to be more knowledgeable due to recent academic exposure and digital literacy. Our findings suggest that while younger individuals may benefit from formal education, practical, on-the-job exposure among older professionals may compensate for a lack of recent training. Effect sizes, while modest, indicate a meaningful trend worth further exploration.

Female participants scored significantly higher than males in domains related to attitudes and help-seeking, aligning with O’Connor and Casey (12) and broader health literature that highlights greater empathy and proactive mental health engagement among women. While the effect size was moderate (Cohen’s d ≈ 0.4), it signals the need for gender-targeted mental health training initiatives, particularly for male healthcare workers, who may under-identify psychiatric symptoms and delay patient referrals.

Education level and income also emerged as significant predictors. Higher educational attainment was associated with better MHL, particularly in symptom recognition and treatment awareness, reinforcing the findings of Kutcher et al. (2). However, this relationship is not linear nor uniform, as illustrated in Elyamani and Hammoud (17) and Marangu et al. (18), who found low MHL even among academically trained professionals in resource-constrained or underserved contexts. Thus, formal qualifications alone may be insufficient without active professional reinforcement, clinical exposure, and training.

Personal exposure to mental illness, whether through patients, family, or personal experience, strongly influenced MHL. This is consistent with Morgan et al. (19), who highlighted the role of familiarity in reducing stigma. Yet, as shown by Oztas and Aydoğan (20), exposure outside structured mental health units may still result in suboptimal literacy unless supported by formal guidance and clinical supervision.

Cultural and systemic influences in Saudi Arabia must also be acknowledged. Despite growing national attention to mental health, cultural stigma, reliance on family-based care, and the limited integration of mental health into primary care continue to act as barriers. Religious beliefs and societal norms may influence provider attitudes, shaping their willingness or confidence in addressing psychiatric disorders.

While the findings are robust, the study is subject to limitations. Self-reported responses may introduce social desirability bias, and the cross-sectional design limits causal inference. Moreover, confounding variables such as specialty area, years of clinical experience, prior mental health training, or type of institution (public vs. private) were not fully controlled. Future research should incorporate multivariate regression models to isolate independent predictors of MHL.

### Future directions

To build upon these findings, the following areas are recommended for further investigation:

1. Multivariable analyses that control for confounders such as clinical experience and workplace mental health training.
2. Longitudinal studies to assess whether MHL improves over time with targeted education.
3. Evaluation of differences in MHL across healthcare settings (urban vs. rural, public vs. private).
4. Development and validation of Saudi Arabia–specific MHL interventions that are culturally and linguistically appropriate.
5. Implementation of interventional studies to assess the impact of structured MHL training on clinical decision-making and patient outcomes.

## Conclusion

The study showed significant associations between MHL and socio-demographic factors among healthcare providers in Saudi Arabia, emphasizing the need for targeted interventions to improve MHL across different groups. One of the steps toward improving health care would be to improve MHL among healthcare workers, which in turn could help advance mental health services—a focus area for Saudi Arabia. In order to achieve this, targeted programs for MHL need to be designed and implemented specifically catered toward male healthcare workers, lower educated, and lower monthly income groups.

## Data Availability

The original contributions presented in the study are included in the article/supplementary material, further inquiries can be directed to the corresponding author.
